# Let’s talk to women, not about them: pregnant women’s perspectives on integrated maternity care in the southwestern region of the Netherlands

**DOI:** 10.1186/s12884-026-09201-2

**Published:** 2026-05-21

**Authors:** Anne C.M. Hermans, Maria A. Hermus, Julia Spaan, Jantien Visser, Vincent J.T. Peters, Maria P.H. Koster, Arie Franx, Jacoba van der Kooy

**Affiliations:** 1 Department of Obstetrics and Gynaecology, Erasmus MC, Rotterdam, Netherlands; 2 Midwifery Practice Oosterhout, Oosterhout, Netherlands; 3 Department of Obstetrics and Gynaecology, Amphia hospital, Breda, Netherlands; 4 Department of Information Systems and Operations Management, ilburg University, Tilburg, the Netherlands; 5 National Institute for Public Health and theEnvironment, Bilthoven, Netherlands

**Keywords:** Integrated maternity care, Women’s perspectives

## Abstract

**Background:**

With the introduction of integrated maternity care in the Netherlands, efforts have focused on enhancing interprofessional collaboration, exemplified by integrated care pathways. Incorporating women’s perspectives is essential for the ongoing development of integrated care pathways. Therefore, this study explores pregnant women’s experiences with integrated maternity care.

**Methods:**

Data collection included twelve in-depth interviews with pregnant women receiving care within an integrated maternity care organisation (IMCO) in the Netherlands, guided by the World Health Organisation domains of responsiveness topics. A reflexive approach to thematic analysis was performed.

**Results:**

We found that pregnant women’s experiences were influenced by (1) care consistency with different views regarding substantive agreement among healthcare providers and care provider consistency; (2) information provision during pregnancy concerning the need for timely and accessible information to make them feel well-prepared for childbirth, and (3) care provider engagement, defined by their approach and the degree of patient-tailored care.

**Conclusion:**

This study offers valuable insights into women’s experiences with integrated maternity care. Women emphasized the importance of both substantive agreement among healthcare providers and continuity of caregiver as essential components of responsive, woman-centred care. Timely and reliable information was also crucial in helping women feel informed, involved, and well-prepared for childbirth. Particularly important for primiparous women, an engaged, personalised approach to care fostered a sense of involvement and control. Strengthening these elements within interprofessional collaboration may enhance the development of integrated care models that are more closely aligned with the lived experiences and needs of pregnant women.

**Supplementary Information:**

The online version contains supplementary material available at 10.1186/s12884-026-09201-2.

## Introduction

In the Netherlands, initiatives to enhance interprofessional collaboration in maternity care were incited by reports of relatively high perinatal mortality rates compared to other Western European countries [1], of which an important contributing factor was the fragmented organization of maternity care.

The Dutch maternity care system is tiered based on the estimated risk of adverse pregnancy outcomes. Primary care for low-risk pregnancies is provided by community midwives; intermediate-risk pregnancies are managed by hospital-based obstetricians in secondary care; and high-risk pregnancies are referred to tertiary care in university medical centres. During pregnancy, it is common for obstetric care providers from both primary care and secondary care to be involved, which can present challenges for the continuity of care [1]. Approximately 80% of pregnant women receive care across multiple tiers, but referrals, particularly from primary to secondary care, can disrupt continuity and lead to the loss of critical medical information.

The Dutch Steering Group on Pregnancy and Childbirth emphasized the need for organisational improvements to enhance the quality of maternity care [1]. They advocated for stronger collaboration between primary and secondary care providers through an integrated maternity care model. In line with this, obstetric care providers in the southwestern region of the Netherlands recognized that limited integration across care levels hindered effective interprofessional collaboration, resulting in fragmented care and dissatisfaction among both patients and providers.

These developments culminated in the establishment of an Integrated Maternity Care Organization (IMCO) in 2016, bringing together a multidisciplinary network of community midwives, hospital staff, and professionals from local maternity care organizations. A central feature of this model is the use of an integrated digital patient record, enabling shared documentation across primary and secondary care providers. This integrated patient record reduces barriers for peer consultation between care providers [2, 3]. 

In addition, integrated care pathways were co-created by professionals from both levels of care to support personalized care and foster continuity of care. These pathways improve care coordination and minimize variability in clinical practices [4, 5]. While community midwives continue to manage all low-risk pregnancies, care for intermediate-risk pregnancies is now provided through shared care between primary and secondary care providers. In this model, community midwives, clinical midwives, and consultant obstetricians assume joint responsibility, which is an approach that contrasts with the prior system, in which intermediate-risk cases were managed solely in secondary care.

Through these efforts value-based maternity care is pursued, which means care is patient-tailored, cost-effective, and associated with equal or improved clinical outcomes [6–8]. Incorporating women’s perspectives is essential, given the positive correlation between patient experience, clinical effectiveness, and safety [9].

We hypothesize that integrated maternity care enhances women’s experiences with obstetric care. The international value of survey-based evaluations in assessing women’s experiences with maternity care is well established [10, 11]. For example, Maimburg et al. [12] demonstrated that participation in structured antenatal programmes led to more positive birth experiences compared to standard care, whereas medical interventions were associated with negative experiences. Similarly, Lewis et al. [13], using a mixed-methods design in Western Australia, highlighted the complexity of maternal satisfaction and the importance of respectful, woman-centred care delivered by competent clinicians. Their findings emphasized how mode of birth and the degree of shared decision-making influenced women’s satisfaction across the childbirth continuum. While the value of survey-based evaluations of maternity care experiences is well recognized, surveys may fail to capture important nuances due to superficial questioning, response bias [14], or reluctance to express dissatisfaction [15, 16].

Therefore, the objective of this qualitative study is to gain insights into the experiences and perspectives of pregnant women regarding integrated maternity care. A richer understanding of these experiences can offer essential guidance for improving clinical practice.

## Methods

### Ethical considerations

Ethical approval to conduct this study was obtained from the regional Medical Ethics Committee Erasmus MC (MEC-2021-0107). The respondents were recruited in person at the outpatient clinic of the IMCO. We obtained written and oral informed consent from all respondents to participate in the study and to publish the results in a scientific medical journal.

### Study design

In this qualitative study we explored pregnant women’s experiences with receiving integrated maternity care within an integrated maternity care organisation in the southwestern part of the Netherlands. The COREQ criteria (Additional file 1) were used as a guideline to report our research [17].

Women were approached for participation if they (1) were 18 years of age or older, (2) fluent in Dutch, (3) if they were in their second or third trimester of pregnancy, and (4) received care within the IMCO. Exclusion criteria included being under the age of 18 and lacking proficiency in the Dutch language. This linguistic criterion was applied to ensure that participants could articulate their experiences in depth and that the interviews could be conducted in the most nuanced manner.

We employed a purposive sampling strategy, in which the researcher selected participants who were expected to provide the most relevant and information-rich perspectives for our study aims [18, 19]. In these integrated pathways are a key feature of Dutch integrated maternity care, and we aimed to explore women’s experiences of care that bridged across these professional and organizational boundaries. This allowed us to focus on the relational and systemic aspects of care that are essential to understanding the implementation and reception of integrated maternity services. At pregnancy intake, women are assigned to a care pathway, which encompasses the full continuum of care, including postpartum care, which is provided by primary care midwives. Maternity care assistants are involved in providing this postpartum care.

We recruited women who received care within common integrated maternity care pathways: ‘obstetric history of caesarean section’, ‘gestational diabetes’, ‘small-for-gestational-age in obstetric history’, ‘obstetric history of postpartum haemorrhage’, and ‘obstetric history of a group B streptococcal infection’. Within these five integrated care pathways, the prenatal consultations are systematically planned, and the various responsibilities of the obstetric care providers (i.e., community midwives, clinical midwives, and consultant obstetricians) are clearly defined.

### Interview guide

The interview guide was developed based on the World Health Organization (WHO) domains of responsiveness. The WHO responsiveness domains concern the degree of patient-tailoredness of healthcare and consists of domains that describe the interaction between care providers and women, and domains that concern the organisation of care [21]. They reflect key aspects of patient-centred care and include respect for the dignity of persons, autonomy in health-related decision-making, privacy and confidentiality, prompt attention, adequate quality of basic amenities, communication, access to social support, and choice of medical providers. Through extensive discussions with supervisors JvdK and MK, we selected three core themes from these universal domains that were most relevant to our research context to include in the interview guide (Additional file 2): (1) autonomy, focusing on women’s involvement in decision-making and the establishment of an equal, collaborative relationship between the pregnant woman and the care provider. (2) communication, addressing whether women felt heard and had sufficient opportunity to ask questions during prenatal care, and (3) information, referring to the clarity and comprehensibility of the information provided to women throughout the care process.

### Data analysis

A reflexive approach to thematic analysis was performed. This approach highlights the researcher’s active role in knowledge production [20]. Researcher AH carried out the thematic analysis of the data by using Atlas.ti software [21]. The software facilitated the coding process, enabling the researchers to devise a coding scheme, apply codes to pertinent text segments, and link related codes together.

First, researcher AH read the transcripts to get familiarized with the data. AH inductively coded the first three interviews, after which she discussed the code scheme with WK and JvdK. These codes were then grouped into broader themes through a process of constant comparison and iterative refinement. To enhance rigour, the coding and interpretation were discussed regularly within the research team, particularly between the first author (AH) and supervisors (JvdK and MK). These discussions ensured that emerging themes were grounded in the data and allowed for critical reflection on potential biases or assumptions. Memo-writing and the use of analytic notes during coding supported this reflexive process. Discrepancies in interpretation were resolved through discussion until consensus was reached. After agreement on the coding scheme was reached, researcher AH coded the remaining transcripts. Throughout the coding process, the analysis, findings, and quality of the quotes were critically evaluated. Thematic saturation was defined as the point at which no new themes emerged from the interviews. In consultation with supervisor JvdK, it was concluded that thematic saturation had been achieved.

Through this combination of inductive coding and team-based reflection, trustworthiness of the findings was pursued. Subsequently, AH created an overview of the derived key themes and subthemes. Please see Additional file 3 for the code tree scheme.

## Results

Initially, twenty-three participants were approached for participation at the antenatal outpatient clinic. These women were often already in the later stages of the third trimester of pregnancy, and this timing influenced their availability for interviews and led to a natural narrowing of the final sample. Eventually, twelve women agreed to take part. Reasons for non-participation included: no response after the invitation, the perceived length of the interview being too long, or start of labour before the interview could take place.

Data were collected by researcher AH through semi-structured interviews. From February to April 2021, 12 online interviews were conducted online via Zoom [22]. The interviews had an average duration of 41 min. Audio recordings were made using the Zoom platform, with both verbal and written informed consent obtained from all participants prior to the interview. Recordings were transcribed verbatim and subsequently anonymised for analysis. In addition, field notes were taken during and immediately after each interview to document contextual details and initial analytical impressions. Due to social restrictions in the Netherlands during the Covid-19 pandemic it was not possible to interview the respondents in person.

Thematic saturation was reached after ten interviews, after which 2 additional interviews were conducted, which confirmed that no new themes emerged.

Table 1 provides the demographic characteristics of the participating pregnant women. Most women were multiparous, Dutch, and had completed a higher level of education.

**Table 1:** Demographic characteristics of the respondents

Respondent’s number for quotes	Age (years)	Parity	Nationality	Marital status	Education level	Care pathway
1	30–34	Multiparous	Dutch	Married	University	Caesarean section in obstetric history
2	35–39	Multiparous	Dutch	Living together with partner	University	Caesarean section in obstetric history
3	30–34	Multiparous	Dutch	Living together with partner	Higher professional education	Gestational diabetes mellitus
4	35–39	Multiparous	Dutch	Living together with partner	University	Small-for-gestational-age in obstetric history
5	35–39	Multiparous	Dutch	Married	University	Caesarean section in obstetric history
6	30–34	Multiparous	Dutch	Married	Higher professional education	Post-partum haemorrhage in obstetric history
7	35–39	Multiparous	Dutch	Living together with partner	University	Caesarean section in obstetric history
8	20–24	Primiparous	Other	Married	Higher professional education	Group B streptococcus carriage
9	35–39	Multiparous	Dutch	Married	University	Caesarean section in obstetric history
10	35–39	Multiparous	Dutch	Registered partnership	University	Small-for-gestational-age in obstetric history
11	30–34	Primiparous	Dutch	Living together with partner	University	Gestational diabetes mellitus
12	35–39	Multiparous	Dutch	Living together with partner	University	Caesarean section in obstetric history

Three key themes were identified based on the experiences and perspectives of pregnant women with received integrated maternity care: (1) Consistency of care, (2) Information provision, and (3) Engagement of care providers.

**Fig. 1:**
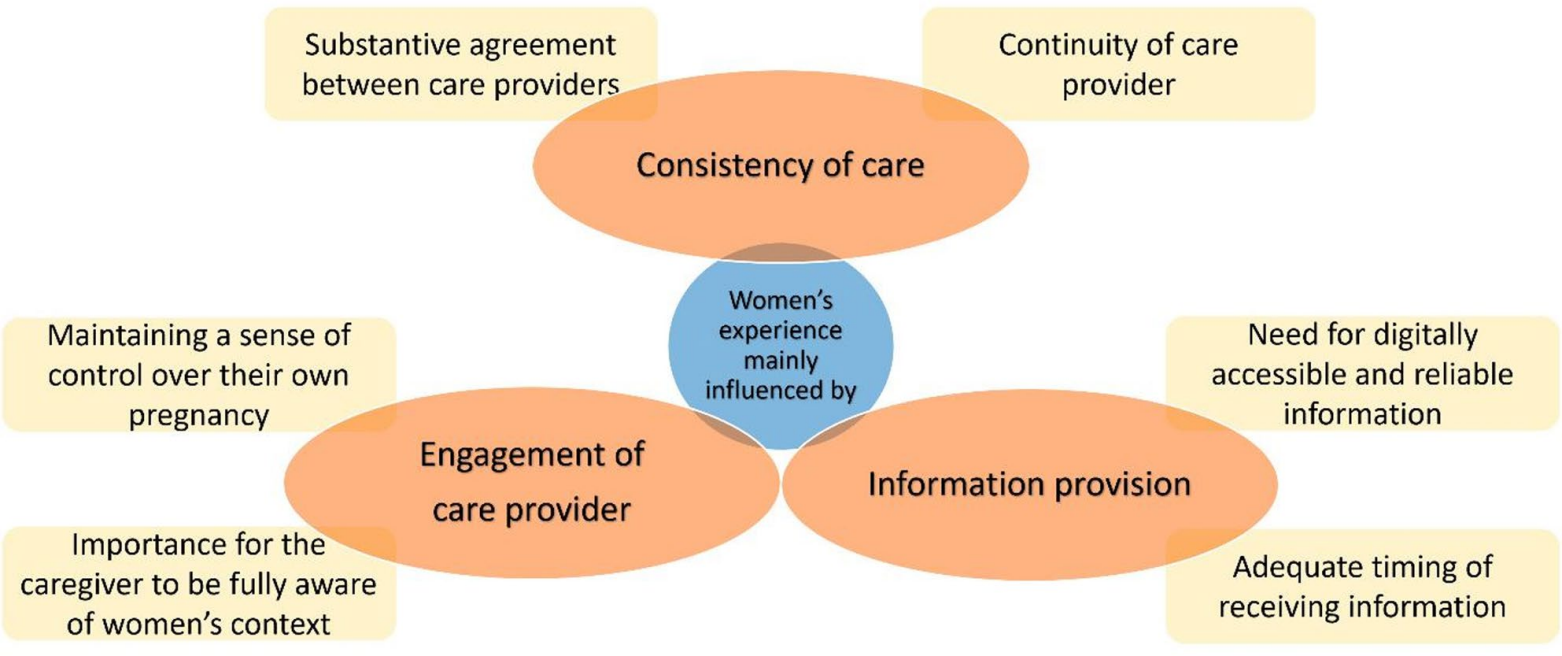
Identified key (sub)themes regarding what influences women’s experience with received integrated maternity care. Orange: key themes, yellow: subthemes

Figure 1 comprises the identified themes and their corresponding subthemes. In the following section these (sub)themes will be discussed with support of relevant quotes of the interviews.

### Theme 1: consistency of care

This theme explores women’s experiences and perspectives on the consistency of care, which is a key aim of integrated care pathways. Although most women were not fully aware of how their care pathway was organised, particularly regarding the collaboration between primary and secondary care providers, they did have a clear understanding of the indications related to their pregnancy and the type of care they were receiving, as well as the reasons for that care.

One respondent mentioned that she is not aware of receiving care according to a specific care pathway:

*R: “No not really. It [the received care] goes smoothly*,* and I feel the provided care of midwives and gynaecologists is intertwined. To me it was not immediately clear that the care was provided through a care pathway. (…).* - Respondent 10.

(…)

*R: “Oh*,* I don’t mind that all. If only the consultations are being planned as they should.”*

#### Continuity of care provider

Within the IMCO, pregnant women could meet different care providers at each prenatal consultation: sometimes a community midwife in primary care, and at other times a clinical midwife or obstetrician in secondary care. While some respondents did not mind seeing different care providers each time, others expressed frustration about the lack of continuity.

One respondent highlighted the confusing and repetitive nature of seeing different obstetricians throughout her care pathway:

*“What I found very annoying is that I saw six different gynaecologists. Different stories*,* different opinions. (…). The file is opened again and again*,* and the same questions are asked.” – respondent 7*.

Another respondent echoed this sentiment and described how this lack of continuity led her to feel unseen and unheard:

*“And then (…) I do think that in a hospital*,* you’re just a number*,* right? And the same goes for the midwife*,* by the way. Honestly*,* I was glad it was my second pregnancy*,* also because of COVID*,* of course. But to be honest*,* I never saw the same person twice. And if you want to advocate a little for how you want things—because my first pregnancy*,* or rather my first delivery*,* wasn’t really a positive experience—then you have to… I genuinely think I had to repeat my story 20 times*,* because I really felt like I had to stand my ground. So I told that story 20 times. And 20 times*,* they made a note of it […].” – respondent 1*.

This quote highlights the respondent’s frustration with the lack of continuity in care providers during her pregnancy. The repeated need to explain her history and preferences created an additional burden. While she referred to seeing different professionals, her core concern lay in not feeling heard or acknowledged—especially considering a previous negative birth experience. Her account underscores the importance of not only consistent care but also genuine attention to the individual needs and preferences of patients.

Five respondents, however, valued seeing different healthcare providers at each prenatal visit, as it reassured them to have met several care providers ahead of an unpredictable delivery team during childbirth.

*“It is also nice to see multiple care providers beforehand so that when I go into labour*,* I am not suddenly faced with a complete stranger. Of course*,* that could still happen*,* but I don’t mind seeing a different person each week in the final weeks.” - respondent 9.*

#### Substantive agreement between care providers

Five respondents expressed that seeing a different care provider at each visit was not problematic, provided that all professionals involved were well-informed and aligned in their approach. For these women, consistency in the content of care through effective communication and shared understanding among providers was more important than continuity with the same individual.

*“That continuous line [is important to me] and that you do not have someone who has the wrong information. That would be such a blooper!” – respondent 4*.

Another respondent expressed appreciation for the substantive agreement among different care providers, highlighting the reassurance it gave her that they were aligned in their approach.

*“I do not have the feeling I have to tell everything over and over again [to different care providers] or that they [the obstetric care providers] miss things.*

*“[For example]*,* early this pregnancy I asked my midwife about [the term to deliver] and when later in pregnancy I discussed the same thing with my gynaecologist*,* they were on the same page*,* which was nice” – respondent 10*.

These experiences suggest that women value different aspects of continuity. While some prioritized seeing the same provider, others found substantive agreement among providers more important. Both forms of continuity contributed to a more positive care experience.

### Theme 2: information provision

This theme encompasses women’s experience and perception towards the written and oral information that was provided during the prenatal visits. Overall, most women experienced the information they received from both the midwife and the care providers in the hospital as uniform and sufficient. Some respondents, however, felt that proactive engagement and self-advocacy were important for obtaining information about their care trajectory.

*“Because you just get […] if you do not ask about it*,* you get very little information from the midwife. Of course*,* you can get a lot from books and such (…) I can imagine if people are a little less assertive in their first pregnancy that they might not get a lot of information or start looking up a lot of information and then maybe not find the right information.” – respondent 10.*

#### Adequate timing of receiving information

Furthermore, four respondents perceived the amount of information as overwhelming and expressed the need for dosed, patient-tailored information throughout their pregnancy.

*“Yes*,* it [the provided information about mode of delivery after a previous caesarean section] was a bit overwhelming. I knew about it*,* but it still caught me off guard—I was only 21 weeks pregnant*,* and suddenly all the risks were being discussed again. Since I already knew what I wanted*,* it didn’t bother me as much*,* but I can imagine it being really overwhelming for someone who wasn’t prepared.” - respondent 9*.

*“(…) do not go too fast*,* because we [pregnant women] always go slower than you think. (…) keep it [the information] as simple as possible: small steps in the provision of information*,* no [information] overload.” – respondent 5*.

#### Need for digitally accessible and reliable information

Throughout the interviews, women mentioned that, especially during their first pregnancy, they tended to search for information about pregnancy online but sometimes struggled to find reliable sources.

*“(…) during my first pregnancy*,* I wanted to know everything about what was happening to my body. I could ask the midwife about any symptoms and whether they were normal*,* but there’s also a lot of information online about different stages of pregnancy*,* which I often looked up on Google.” - respondent 2*.

*“(…) Parenting forums often have too many conflicting opinions*,* but this app [I use] seems to provide accurate and trustworthy information.” – respondent 12*.

### Theme 3: engagement of care provider

This theme comprises women’s perception of engagement of the obstetric care provider (i.e., community midwife, clinical midwife, or obstetrician). Engagement of healthcare providers means the provider is not only aware of the woman’s medical situation and preferences during childbirth, so maternity care can be provided in a patient-tailored approach.

*“The midwifery practice is small-scale. I understand that a hospital is larger including more people (…) but the most important thing I thought was that the person who supervised my childbirth was well prepared. She [the midwife in secondary care] knew what I wanted [during childbirth] and what was important to me*,* and she included my birth plan (…) even though I never spoke to her before*,* it made me feel that she took me very seriously and my preferences were really considered.” (…) – respondent 4.*

#### Importance for the care provider to be fully aware of a woman’s context

Four respondents, including respondent 12, emphasized the importance of caregiver engagement, noting that when obstetric care providers are engaged, it enhances their confidence and trust in the care they received.

*“(…) If you see how involved*,* for example she [the consultant obstetrician] is*,* I really thought*,* just heart-warming so to speak and very nice*,* that I felt that she really knew who I was. That is just very nice*,* and I have every confidence in her.”* - respondent 12.

#### Maintaining a sense of control over their own pregnancy

Furthermore, multiple respondents emphasized the importance of feeling that they had a choice, which positively influenced their pregnancy and birth experience. Feeling encouraged to ask questions played an important role in this.

*“With my first pregnancy*,* we just went along with what the medical team advised and never really thought about the fact that I had a choice. A lot happened*,* but I never asked myself: Is this what I want? Now*,* I feel more confident because the medical team is open to my questions and decisions. They obviously have more expertise*,* but I like that my choices are respected and that I can ask as many questions as I need to make an informed decision. (…) I like having control*,* and my partner is on board with that. It’s important to be able to ask questions like ‘What if we wait?’ and to be involved when doctors suggest something. I really want to have some control over the process.” – respondent 7*.

*R: “Yes*,* [the guidance now better meets my expectations] than during my very first pregnancy*,* which was a breech presentation. At that time*,* I had the choice between a C-section or not. I actually expected them to tell me*,* ‘This is the best option; you should choose A.’ But of course*,* that’s not how it works—you ultimately have to make the decision yourself. Now that I’ve gone through several pregnancies*,* I’ve also become wiser*,* and I view it differently than I did the first time. You actually have a lot of autonomy; you have a great deal of choice. During my first pregnancy*,* I expected them to simply tell me*,* ‘Well*,* the best option for you is this or that*,*’ because that’s what I thought would happen.” – respondent 5*.

## Discussion

The aim of our study was to gain insight in pregnant women’s experiences and perspectives of integrated maternity care. Our findings indicate that pregnant women’s experiences with integrated maternity care were influenced by (1) care consistency with different views regarding substantive agreement among healthcare providers and care provider consistency; (2) information provision during pregnancy concerning the need for timely and accessible information to make them feel well-prepared for childbirth, and (3) care provider engagement, defined by their approach and the degree of patient-tailored care.

The participants presented a diverse range of experiences and perspectives regarding the maternity care they received. Overall, women expressed positive sentiments about their received maternity care. However, upon further exploration, varying views emerged regarding the factors they considered important for a positive pregnancy experience, which in itself highlights the need for models of care that can accommodate individual preferences and expectations. A key insight that became evident is that, in maternity care, there is no ‘one size fits all’ model. However, an Integrated Maternity Care Organization (IMCO), characterized by intensive interprofessional collaboration, could facilitate the coordination of care while striving toward patient-tailored maternity care. In doing so, it could incorporate the following themes to enhance the maternity care experience for expectant and new mothers.

### Consistency of care

Regarding the first key theme, participants highlighted the importance of different dimensions of continuity. While some valued continuity with the same care provider, others placed greater emphasis on substantive consistency across providers. These findings suggest that women differentiate between relational and informational continuity, with both contributing to a more positive care experience. Overall, substantive agreement between care providers with consistent information was considered equally, if not more, important than being seen by the same provider throughout. Previous literature presents different views on consistency of care, and consistency of care provider. The study of Brady et al. [23] found that the consequences of continuity of care, rather than continuity of care itself, such as shared decision-making and improved outcomes are linked to women feeling empowered to navigate their care trajectory. Furthermore, antenatal continuity of care, for example provided within a midwife-led model of care, is associated with reduced prenatal hospital admissions with the most significant impact on clinical outcomes occurring during the intrapartum period [24]. However, some studies caution against confusing continuity of care with continuity of care provider. While these concepts are related, it is possible to achieve care satisfaction and continuity of care without a single, fixed provider. ‘Continuity of care’ involves seamless, coordinated care supported by effective communication and consistent policies across all care providers. In contrast, ‘continuity of care provider’ specifically refers to the number and consistency of care providers [25]. Despite the ongoing debate in the literature regarding the importance of care provider continuity [26], we pose that the true value lies in the consistency and quality of provided care – specifically having a care provider that is familiar with the woman’s preferences capable of delivering high-quality care, emphasizing substantive consistency [25, 27, 28]. Previous literature demonstrated that having met an obstetric caregiver during pregnancy is less important for a positive birth experience than receiving continuous care from the same caregiver during labour [25, 29]. Therefore, we suggest that the focus should lie on the overall quality of a woman’s care experience. This includes ensuring that women receive clear and consistent guidance, have timely access to necessary support, feel well-informed, and feel a sense of control over their care, without experiencing deficiencies in service delivery [4, 25, 30]. An important asset in realizing consistency of care are integrated care pathways, which stimulate organised and efficient prenatal care, help to reduce the variability in clinical practice, and improve clinical outcomes [4, 31]. Additionally, working with an integrated patient record allows care providers from both primary and secondary care to review each other’s records and receive the necessary information to provide patient-tailored care. A meta-synthesis of qualitative research on patients’ perspectives on care continuity has emphasized on the role of health records in ensuring informational continuity of care [32]. Last, to enhance substantive agreement between healthcare providers, integrated training sessions that incorporate feedback from women and allow them to learn from each other while reflecting on their own counselling practices can be effective to improve care consistency, and ultimately improve the quality of care [33–35]. 

### Information provision

During pregnancy, women receive information at predetermined times according to their care pathway. For most women, this was perceived as adequate, although some women felt the information was not tailored to their specific situation. They expressed the need for simple, patient-tailored information at the right time during pregnancy. This aligns with existing literature on women’s prenatal care experiences regarding health education in maternity care [36]. 

Furthermore, some women indicated that the amount of information provided during pregnancy was occasionally overwhelming and not always delivered at a time that aligned with their needs or space to process it effectively. Previous literature on midwives’ efforts to provide patient-tailored information underlined its importance but also highlighted the challenges of adapting information to a woman’s health literacy level [37]. Developing a technical innovation, like a pregnancy app, is one potential way to tailor information to women. The introduction and implementation of such an innovation could be easily achieved within an IMCO because of the strong interprofessional collaboration that already exists. Previous literature shows that digital services have empowered both healthcare providers and women, thereby increasing engagement [38]. Furthermore, as mentioned in the interviews, an app with reliable information about their pregnancy could enhance women’s experience of maternity care.

### Engagement of care provider

Pregnant women consistently emphasized the importance of healthcare providers being fully aware not only to their medical status but also to their individual needs and preferences [39]. This was particularly relevant for primiparous women, who often require more reassurance and guidance. A personalised and engaging approach to care fostered a sense of involvement and control, especially when women were encouraged to ask questions and actively participate in decision-making. Although most participants in our study were multiparous, several reflected on their first pregnancy and described a previously lacking sense of autonomy, which they felt had improved through experience in subsequent pregnancies.

According to previous research, caring for women in a respectful approach was seen as the essential healthcare priority for women worldwide [40, 41]. Another study on what enhances respectful maternity care emphasized on the importance of acknowledging the woman’s broader social context and past experiences [42]. In situations where maternity care was fragmented, or in case of a lack of adequate communication, the quality of women’s experience with maternity care was negatively affected [39]. In line with previous literature, the value of experiencing high quality of care lies in the *way* women are cared for rather than just the fulfilment of specific birth preferences [43, 44]. 

### Strengths, limitations, and suggestions for future research

By using a qualitative research design, we were able to delve deeper into the women’s perspective on their received maternity care. Our qualitative research therefore complements quantitative research, which often lacks the nuance needed to fully capture participants’ lived experiences. A key strength of the qualitative approach lies in its capacity to uncover underlying needs, motivations, and expectations that may remain hidden in standardized survey-based studies. Qualitative methods allow for a contextualized understanding of how care is experienced and interpreted by patients, which is essential when aiming to improve patient-centred care [45].This approach helped us illustrate not only what women experienced, but also how and why those experiences held meaning for them within the broader context of integrated care.

It is important to acknowledge the limitations of our sample. Most participants were multiparous, and all participants were Dutch and highly educated. We selected participants who were receiving care through maternity care pathways that involved both primary and secondary care providers (‘shared care’). Since the indication for shared care was related to obstetric history, most participants were multiparous and slightly older in age. As a result, perspectives from more diverse sociocultural and educational backgrounds, as well as those of first-time mothers, may be underrepresented. Furthermore, women were approached at the antenatal outpatient clinic and were often already well into their third trimester at the time of inclusion, as they had by then experienced most of the care pathway. Consequently, many participants became unavailable for interview due to increasing physical demands of late pregnancy, imminent delivery. Future research should aim to include a broader demographic to further inform the development of inclusive and equitable integrated care models. Furthermore, to expand international knowledge on what women consider important regarding their maternity care experience, future studies should be conducted in different cultural and national contexts where maternity care is organized differently.

## Conclusion

This study offers valuable insights into women’s experiences with integrated maternity care. Women emphasized the importance of both substantive agreement among healthcare providers and continuity of caregiver as essential components of responsive, woman-centred care. Timely and reliable information was also crucial in helping women feel informed, involved, and well-prepared for childbirth. Particularly important for primiparous women, an engaged, personalised approach to care fostered a sense of involvement and control. Strengthening these elements within interprofessional collaboration may enhance the development of integrated care models that are more closely aligned with the lived experiences and needs of pregnant women.

## Supplementary Information

Additional file 1.

Additional file 2.

Additional file 3.

## Data Availability

No datasets were generated or analysed during the current study.
