# Supplementary material for: Let’s talk to women, not about them: pregnant women’s perspectives on integrated maternity care in the southwestern region of the Netherlands
Source: BMC Pregnancy Childbirth. 2026 May 21;26:784. doi: 10.1186/s12884-026-09201-2 (PMC13377813; doi:10.1186/s12884-026-09201-2)
Supplement: Supplementary file 2 — Additional file 2. [file 12884_2026_9201_MOESM2_ESM.docx]

**Appendix B. - Example of an interview guide used during the interviews.
*These questions are care pathway specific. In this case, the evaluated care pathway was having a caesarean section in obstetric history.**

| Section | Question | Probes |
| --- | --- | --- |
| Introductory Question | How do you look back on the course of your pregnancy? | *What stands out the most for you?* |
| Care Path Evaluation* | In this interview, we aim to evaluate (and improve) the current care pathways. |  |
|  | How did you experience this care pathway? | *What aspects did you find effective?*  *Were there any areas that could be improved or that you found challenging?* |
|  | During this pregnancy, your preferred method of delivery (caesarean or vaginal) was discussed twice. What did you think of this counselling? | *Was the frequency of discussions appropriate (too few, too many, too early, or too late)?* |
| Autonomy | To what extent did the support during your pregnancy meet your expectations? | *Why?  Were there aspects you missed? What did you appreciate about the support?  Were there any aspects you found less effective?* |
|  | To what extent did you feel in control of your pregnancy? | *Can you elaborate?  Were there moments you wanted more control?  Were there moments where you felt care providers took over too much?* |
|  | Do you feel you were involved in decisions about tests or treatments during your pregnancy? | *How were you involved in decision-making?  Are there moments where you would have liked to contribute more?* |
|  | Did you feel confident about the delivery? | *Why?* |
| Communication | How did you find the way the healthcare provider (e.g., doctor or midwife) informed or explained things during consultations? | *What did you find effective? What could have been improved?* |
|  | Was there space during consultations to ask questions if you didn’t fully understand something? | *Can you provide an example? Was there enough room to ask questions?* |
|  | Did you feel you had a designated point of contact during your pregnancy? | *What did you think of having a dedicated contact person?  Did you miss having one?* |
| Information | Did you feel well-informed in general during your pregnancy? | *Why?  Do you feel there was any information you missed that you would have liked to know in hindsight?* |
|  | Did the information provided by your doctor meet your expectations? | *Can you provide an example?* |
|  | Did the information provided by your midwife meet your expectations? | *Can you provide an example?* |
|  | Did you seek information from your close contacts (e.g., partner, friends, family) when you had questions about your pregnancy? | *When did you do this?  What kind of information did you seek from them?* |
|  | Did you use (digital) sources for information about your pregnancy (e.g., for physical complaints)? | *Which sources did you use (e.g., brochures, videos, courses, websites)?* |
| Closing Question | Looking back on your pregnancy, what would you like to say to the healthcare providers? | *Do you have any tips or final thoughts? Is there anything you would like to revisit?* |
|  | Thank you for your time! |  |
