# Supplementary material for: Let’s talk to women, not about them: pregnant women’s perspectives on integrated maternity care in the southwestern region of the Netherlands
Source: BMC Pregnancy Childbirth. 2026 May 21;26:784. doi: 10.1186/s12884-026-09201-2 (PMC13377813; doi:10.1186/s12884-026-09201-2)
Supplement: Supplementary file 3 — Additional file 3. [file 12884_2026_9201_MOESM3_ESM.docx]

| Main theme | Subtheme | Illustrative/Exemplary quotes |
| --- | --- | --- |
| 1. Consistency of care | Substantive agreement between caregivers | *"Overall, the consultations were very pleasant and largely consistent. The information provided was generally aligned, and the consultations were quite similar, allowing me to know what to expect, which was reassuring."* ***[respondent 1]***  *"I do notice differences depending on which midwife I see. Some take a more pragmatic approach, while others are more intuitive and take a more wait-and-see approach. However, I feel that, overall, there is a good balance within the team."* ***[respondent 12]***  *"I don’t really notice that I’m in a care pathway. The collaboration between the midwife and the obstetrician flows quite smoothly, and their roles overlap. It wasn’t immediately clear to me that there was a set care plan in place****." [respondent 10]***  *“Yeah, I don’t really mind that it’s a different person each time, as long as they keep things somewhat consistent.”* ***[respondent 3]*** |
|  | Continuity of caregiver | *"You never know who will be at your bedside, so in that sense, it might be preferable to have a higher chance of seeing someone familiar. While it may be reassuring to see the same person each time, I personally don’t find it that important."* ***[respondent 10]***  *"Of course, there is always the issue—depending on the midwifery practice you are with—that you may see some midwives more frequently than others. It could happen that a midwife you have only met once ends up being the one attending your birth. I might not find that very appealing, but at the same time, I feel that in the moment, it probably wouldn’t matter much."* ***[respondent 12]***  *"I think it helps build a connection, and having a regular caregiver feels more like a steady support than constantly seeing someone new."* ***[respondent 7]***  *"Well, you know, it’s actually kind of nice to see different people – that way, when I go into labor, it won’t be a complete stranger by my side. I mean, that could still happen, of course, but I don’t mind seeing someone different every week in the final stretch.* ***[respondent 9]*** |
| 2. Information provision | Adequate timing of receiving information | *"I think it’s good that the topic [counseling about mode of delivery after having had a caesarean in obstetric history] was discussed twice. If it had only come up once early in pregnancy, it would have felt too soon, as your perspective changes over time. But at the same time, I do think it’s important to bring it up early, so you have time to think about it and it doesn’t suddenly come as a surprise later on."* ***[respondent 5]***  *"The timing [of information provision] was fine since I knew a handover would happen at some point. But there was a long gap between appointments, and maybe not enough was covered. Your perspective can change during pregnancy, so more discussions might have been helpful."* ***[respondent 2]***  *"Yes, it [the provided information about mode of delivery after a previous caesarean section] was a bit overwhelming. I knew about it, but it still caught me off guard—I was only 21 weeks pregnant, and suddenly all the risks were being discussed again. Since I already knew what I wanted, it didn’t bother me as much, but I can imagine it being really overwhelming for someone who wasn’t prepared."* ***[respondent 9]*** |
|  | Need for digitally accessible and reliable information | *"I don’t feel that way as much now, but during my first pregnancy, I wanted to know everything about what was happening to my body. I could ask the midwife about any symptoms and whether they were normal, but there’s also a lot of information online about different stages of pregnancy, which I often looked up on Google."* ***[respondent 2]***  *"What I like about [pregnancy app] is that it feels like a reliable source—or at least, I hope it is.* *Parenting forums often have too many conflicting opinions, but this app seems to provide accurate and trustworthy information."* ***[respondent 12]***  *"There’s like 101 things out there, and yeah, I’m not really the type to go looking everything up online. I can usually hold myself back. A lot of the stuff you find isn’t even accurate anyway, so I’d rather hear it from a friend – or a couple of friends – than look it up on the internet****. [respondent 9]*** |
| 3. Engagement of care giver | Importance for the caregiver to be fully aware of women’s context and preferences | *"I really felt like she [the obstetric caregiver] listened to me. Since I was having an induction and my pregnancy was medical, the process was already set. But she still asked if I had any preferences and told me to share them so they could adjust things as much as possible to make the experience better for me. That made me feel really good (…)”* ***[respondent 3]***  *"I like it when caregivers take their time with patients and give them the chance to ask questions. I also appreciate a more personal approach."* ***[respondent 5]***  *"It felt good not to be just another patient in front of a doctor, but someone they actually remembered. When they mentioned past events, it showed me that they knew who I was, which was really reassuring and made me trust them more."* ***[respondent 12]*** |
|  | Maintaining a sense of control over their own pregnancy | *"I would like to have more control, though I know that’s not always easy. The first time, I made a birth plan for my ideal delivery, but it ended up being useless. Now, I still make one, but it is more about communication, getting explanations, and knowing my options rather than insisting on a specific way of giving birth. I understand things might change, but I want caregivers to know how I might react and help me make the best choices, even if my plan needs to be adjusted."* ***[respondent 12]***  *"With my first pregnancy, we just went along with what the medical team advised and never really thought about the fact that I had a choice. A lot happened, but I never asked myself: Is this what I want? Now, I feel more confident because the medical team is open to my questions and decisions. They obviously have more expertise, but I like that my choices are respected and that I can ask as many questions as I need to make an informed decision. (…) I like having control, and my partner is on board with that. It’s important to be able to ask questions like ‘What if we wait?’ and to be involved when doctors suggest something. I really want to have some control over the process."* ***[respondent 7]***  *"This time, I felt more confident—I went in prepared, knowing what I wanted. But during my first pregnancy, when doctors or medical staff gave me advice, I just assumed that was the way it had to be. I never questioned it. One gynecologist kept telling me, ‘You do have a choice,’ and I thought, wow, that’s really nice to hear. Otherwise, advice from doctors can sometimes feel like a set rule instead of something you can talk about."* ***[respondent 1]*** |

**Supplementary table 1. Data coding tree**
